# CO_2_ Mediated Interaction in Yeast Stimulates Budding and Growth on Minimal Media

**DOI:** 10.1371/journal.pone.0062808

**Published:** 2013-04-26

**Authors:** Ilya V. Volodyaev, Elena N. Krasilnikova, Ruslan N. Ivanovsky

**Affiliations:** Department of Microbiology, Faculty of Biology, Moscow State University, Moscow, Russia; Institute of Microbiology, Switzerland

## Abstract

Here we show that carbon dioxide (CO_2_) stimulates budding and shortens the lag-period of *Saccharomyces cerevisiae* cultures, grown on specific weak media. CO_2_ can be both exogenous and secreted by another growing yeast culture. We also show that this effect can be observed only in the lag-period, and demonstrate minimal doses and duration of culture exposition to CO_2_. Opposite to the effects of CO_2_ sensitivity, previously shown for pathogens, where increased concentration of CO_2_ suppressed mitosis and stimulated cell differentiation and invasion, here it stimulates budding and culture growth.

## Introduction

Cell-cell interactions in microbial cultures have been under particular interest and investigation for more than 80 years. The whole area includes works on the so called “mitogenetic effect” [1], [2], “quorum sensing” [3], [4], and rather recently discovered NH_3_ signaling in yeast [5] and CO_2_ sensitivity both in prokaryotes [6], [7] and fungi [8], [9].

The “mitogenetic effect” consists in a distant stimulation of mitosis in prokaryotic and eukaryotic cells by optical contact with other well growing cultures. The effect was shown for bacterial cultures [10], yeast [11], etc., and ultraweak ultraviolet luminescence was stated to be the mediator of these cell-cell interactions [12], [13]. Altogether several hundred articles and monographies appeared in this area, mostly in 1920–1950s, both verifying [14]–[16] and refuting [17], [18] original results. Still, the problem of mitogenetic effect remains unsolved till nowadays.

The effects of chemical cell-cell interactions in microbial cultures, most of them denoted by the notion “quorum sensing”, are proved much more unequivocally. The “quorum sensing” phenomenon (the name given in [19]) consists in cooperative “behavior” of microbial cultures depending on the population density and composition, and including gene expression, cell differentiation, antibiotic secretion, and various virulence-dealing processes, such as hyphae, biofilm and spore formation, and substrate invasion. The mechanism lies in simultaneous secretion and reception of certain species-specific or more or less universal chemicals (small peptides, alcohols, ethers etc.), which accumulate in the medium and switch on certain intracellular signaling pathways when reaching a certain concentrational threshold (for reviews see [3], [4], [20], [21]). Besides these specific signaling factors, cell interaction can be mediated by such a “simple” molecule as NH_3_ [5], which is “used” to synchronize cell differentiation and general morphology of neighboring colonies [22] and prevent them from spreading too close to each other [5].

Can CO_2_ also be a factor of cell-cell interaction? CO_2_ sensitivity of mammalian cells has been known for nearly 50 years [23] and investigated in detail [24]. It has also been shown for cyanobacteria [7], and pathogenic fungi [25], in which it plays the role of “host tissue sensor”. But there are practically no works dealing with CO_2_-mediated cell-cell interaction [9], especially in non-pathogens (discussion of some doubtful data [26] see below). In this work a new case of yeast cell-cell interaction was shown, and the mediator of this was proved to be CO_2_. Thus the observed effect turned out a new case of CO_2_ sensitivity in microorganisms, and a new type of CO_2_-mediated processes, where cell cycle is stimulated rather than suppressed “in favor” of cell differentiation, as it had been well shown for pathogens before.

## Materials and Methods

### Strains

#### Saccharomyces cereviseae

wild-type diploid wine strain VKM J-542 (from the collection of Microb. Dep., Fac. of Biology, Moscow State University).

### Culture Preparation and Media

Suspension cultures were grown on a rotary shaker (120 r.p.m., 30°C) in standard YPD medium (glucose 2%+yeast extract 2%+bactopeptone 1%) till the beginning of stationary phase (18–24 hours), and plated on Petri dishes (1–2×10^8^ cells per 9 cm Petri dish) with agar medium of various composition:

1. rich growth medium – YPD: glucose 2%+yeast extract 2%+bactopeptone 1%+agar 3%, pH5,5;
2. minimal medium with glucose: glucose 0,1%+(NH_4_)_2_SO_4_ 0,1%+KH_2_PO_4_ 0,1%+MgSO_4_ 0,05%+CaCl_2_ 0,01%+NaCl 0,01%+agar 3%, pH5,5;
3. minimal medium with acetate: CH_3_COONa 0,1%+(NH_4_)_2_SO_4_ 0,1%+KH_2_PO_4_ 0,1%+MgSO_4_ 0,05%+CaCl_2_ 0,01%+NaCl 0,01%+agar 3%, pH5,5.

The plated cultures were cultivated at 30°C.

### Measurement

Culture density and budding were measured during experiments.

To evaluate density of agar culture, it was carefully washed off the plate with three portions of water, and optical density of the resulting suspension was measured with nephelometer at 650 nm (OD_650_).

**Figure 1:**
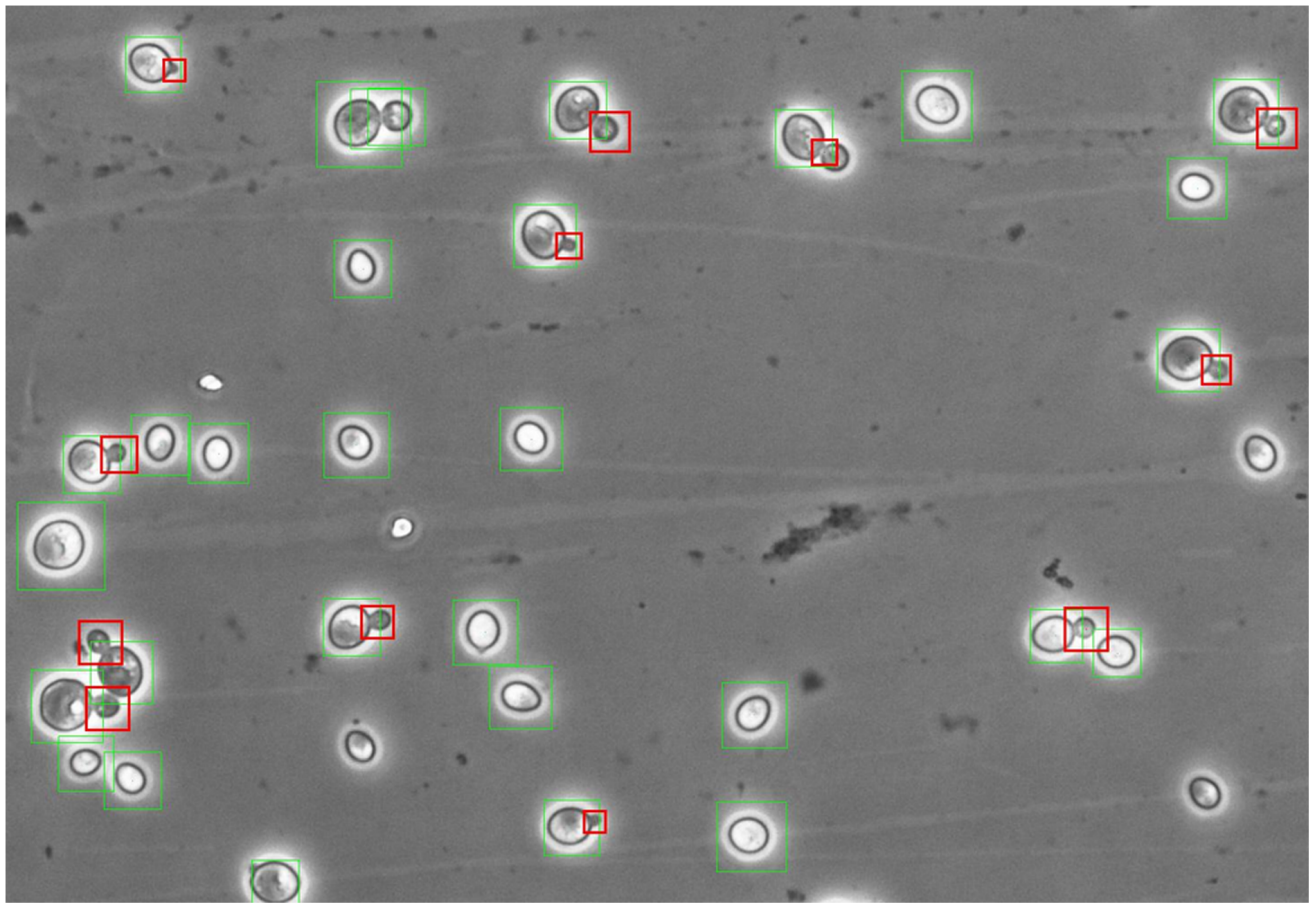
Digital phase-contrast picture of yeast cells. Cells and buds are automatically recognized with a specially constructed software [27]. Recognized cells are marked with green squares, recognized buds – with red squares. Cells smaller than the threshold set in the program, are skipped.

Culture budding was characterized with budding index (BI) – total number of buds divided by the total number of cells counted, in %. To calculate BI of the culture, agar sections of 4 cm^2^ in area were cut off the plates, fixed with formalin, and microphotographed with a phase contrast microscope with 40× objective and digital 5 MP camera. The photographs were digitally processed with specially created original software [27], automatically recognizing cells and buds in digital pictures (fig. 1). No less than 1500 cells were counted for each BI calculation.

**Figure 2:**
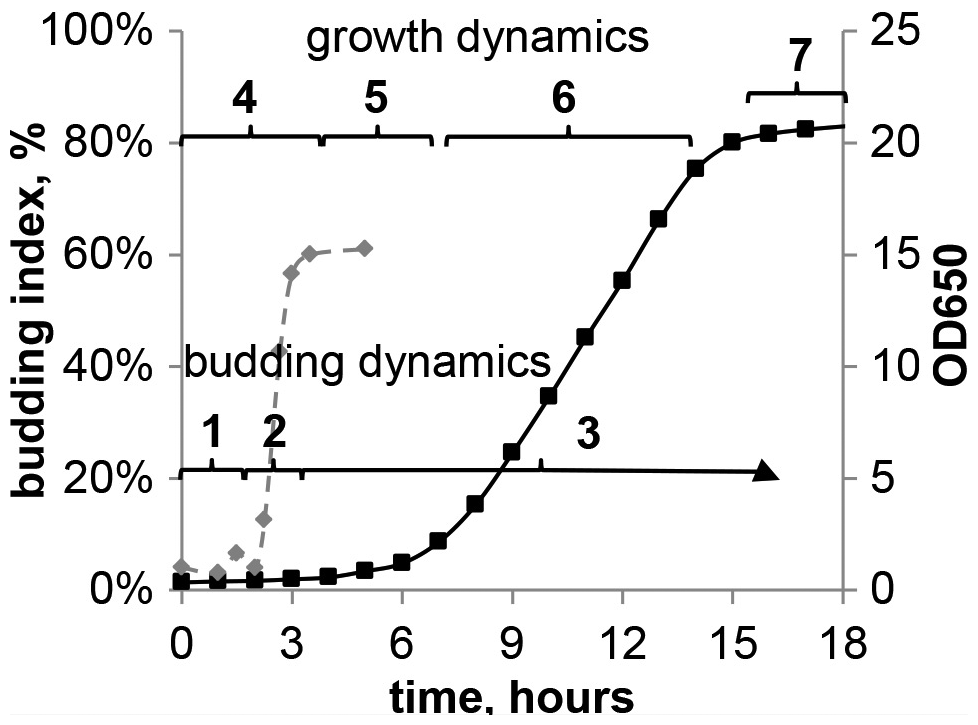
Growth and budding dynamics of *S. cereviseae* on YPD-agar medium. 1– lag period of budding 2– emergence of first buds. Budding index (BI) increase 3– constant BI 4– lag period of growth 5– exponential phase 6– linear growth 7– beginning of stationary phase.

Both culture density and budding were measured periodically, to obtain growth and budding dynamics of the culture (fig. 2).

### Experiment

#### The “induction” experiment

**Figure 3:**
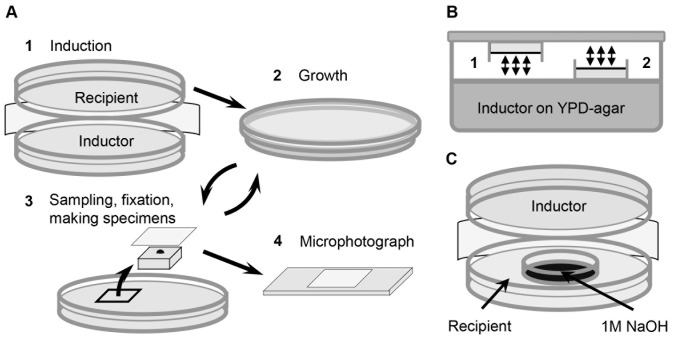
Scheme of experiments. A. The “induction” experiment. 1– Open plates with the “recipient” and the “inductor” yeast cultures were fixed together, their cell layers directed towards each other (1 cm distance between the cell layers), made airtight with parafilm, and left at 30°C for 10–150 min. 2– After that, the inductor was removed, and the recipient was left in a closed dish for further growth. 3– Agar sections of 4 cm^2^ in area were periodically cut off the recipient plate, every 15–30 min, during the first 3–4 hours after the end of induction. Fixing fluid (glycerol : formalin : water, in proportion 50∶ 25 : 25) was spread onto the surface of these samples, imprinted on the cover glass, put on the slide, and… 4– microphotographed with a phase contrast microscope with 40× objective and digital 5 MP camera. B. Testing, whether the inducing factor is a volatile chemical. Hermetically closed airtight container is half filled with YPD-agar and plated with the “inductor” yeast culture. Two open Petri dishes with identical recipient cultures are fixed inside the container. Both recipients are equally available to volatile chemicals (possibly secreted by the inductor). Plate #1 has direct optical contact with the inductor, plate #2 is turned off the inductor and has no direct optical contact with it. Arrows show possible gas exchange. C. Induction in the presence of alkaline trap for (possibly) CO_2_ (1M NaOH) in a small Petri dish, fixed inside the plate with the recipient.

Two open plates with yeast cultures were fixed together, their cell layers directed towards each other, and left for 10–150 min (fig. 3A). After that, one of the plates (called “inductor”) was removed, and the other one (called “recipient”) – closed, and left at 30°C for further growth. The recipient density and budding were periodically gauged, every 15–30 min for 3–5 hours, and compared to budding and culture density in separated single control plates with identical medium and culture.

#### The inducing factor testing

To test whether the inducing factor was a volatile chemical, two identical plates with the recipient culture were fixed inside a big airtight container (20×20×4 cm) half filled with agar medium, and plated with inductor (fig. 3B). Both recipients were equally available to volatile chemical factors from the inductor (arrows in fig. 3B show possible gas exchange), and while one of the recipients had normal optical contact with the inductor, the other one was turned away from it.

In fig. 3C a modification of the standard induction experiment is shown. A small Petri dish with 1M NaOH was fixed inside the recipient plate to partially absorb CO_2_ from the atmosphere inside.

#### CO_2_ inducing experiments

To check the inducing capacity of CO_2_, recipient cultures were put into hermetically closed containers (20×20×4 cm), and atmosphere with various concentrations of CO_2_ (0,1–4%) was created inside by injecting the needed volume of 99,99% CO_2_ into the container, through an airtight rubber stopper.

### Reproducibility

Altogether more than 500 budding curves were registered at various conditions: media content, age of the recipient and inductor cultures, and duration of induction. Each point on the budding curve was obtained by automatic counting of 1500–2000 cells in microphotographs. Each particular experiment was repeated no less than 7 times; some experiments were repeated up to 20 times.

The main experimental data obtained in our work, were budding curves of yeast cultures, which (although looking like standard S-shaped functions), could not be correctly approximated by functions of a single family. Thus we preferred to compare values of budding index in individual time points on the curves. According to our experimental scheme, each experiment had its own control, and thus criteria for dependent samples could be used. As not all the data were always distributed normally, we preferred to use nonparametric Wilcoxon *T-test* for dependent samples to calculate the data confidence. Intervals given in tables are effective 99% confident intervals calculated from normal approximation.

## Results

### 1. Distant Stimulation of Budding and Growth

The “induction” experiment, as shown in fig. 3A, was performed under various conditions – age of the inductor and the recipient cultures, their medium content, and the induction length. Under particular conditions (see below) the experiment led to stimulation of budding and growth in the recipient culture (compared to adequate control).

The main conditions for the induction effect are listed below.

#### The “recipient” cultures can be stimulated only on weak media

**Figure 4:**
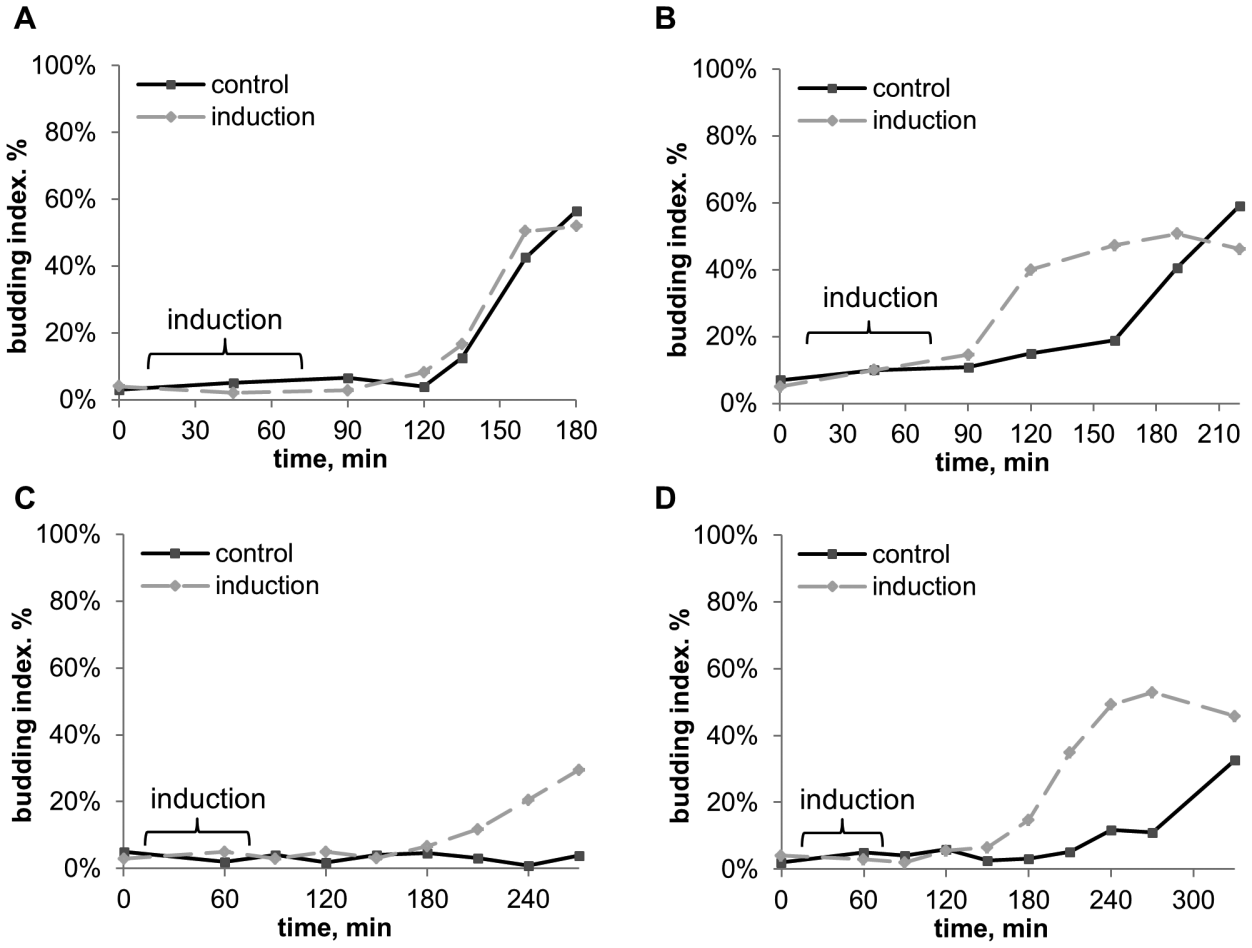
Budding dynamics of *S.cerevisiae* after induction and in control, on various agar media. A. rich growth medium – YPD (glucose 2%+yeast extract 2%+bactopeptone 1%+agar 3%). B. minimal medium with lowered glucose content (glucose 0,1%+(NH_4_)_2_SO_4_ 0,1%+KH_2_PO_4_ 0,1%+MgSO_4_ 0,05%+CaCl_2_ 0,01%+NaCl 0,01%, pH5,5). C. minimal medium with acetate (CH_3_COONa 0,1%+(NH_4_)_2_SO_4_ 0,1%+KH_2_PO_4_ 0,1%+MgSO_4_ 0,05%+CaCl_2_ 0,01%+NaCl 0,01%, pH5,5). D. minimal medium with acetate, without nitrogen (CH_3_COONa 0,1%+KH_2_PO_4_ 0,1%+MgSO_4_ 0,05%+CaCl_2_ 0,01%+NaCl 0,01%, pH5,5). The experiment scheme is given in fig. 3A. Duration of induction was 60 min, inductor – *S.cerevisiae* culture on YPD-agar in early stationary phase (20 hour old), recipient – *S.cerevisiae* culture 15 min after inoculation. No effect is observed on YPD (medium A). On minimal media (B–D) induction leads to BI increase, highly reliable in certain time points: B –120–165 min, *P*<10^−3^; C –210 min, *P*<10^−3^, 240–270 min, *P*<10^−5^; D –180–270 min, *P*<10^−6^.

Plated onto rich growth media (YPD or 2–18° beer worth), the recipient cultures didn’t react to induction, i.e. both budding and growth dynamics of the “induced” culture coincided with control (fig. 4A, growth not shown).

**Figure 5:**
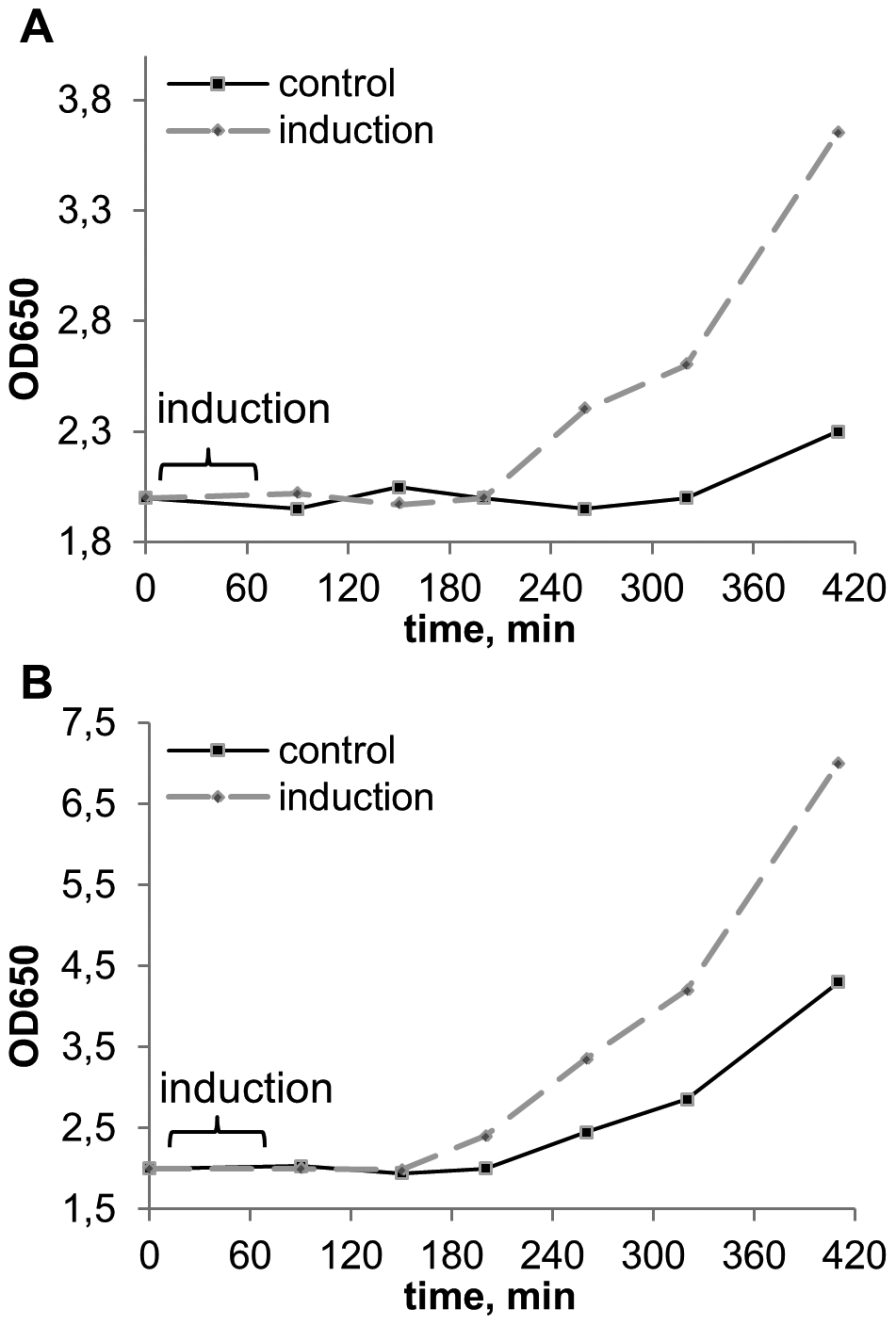
Growth dynamics of *S.cerevisiae* after induction and in control on minimal agar media with acetate (A) and glucose (B) as the main substrate. The medium content: A. CH_3_COONa 0,1%+(NH_4_)_2_SO_4_ 0,1%+KH_2_PO_4_ 0,1%+MgSO_4_ 0,05%+CaCl_2_ 0,01%+NaCl 0,01%, pH5,5. B. Glucose 0,1%+(NH_4_)_2_SO_4_ 0,1%+KH_2_PO_4_ 0,1%+MgSO_4_ 0,05%+CaCl_2_ 0,01%+NaCl 0,01%, pH5,5. The experiment scheme is given in fig. 3A. Duration of induction was 60 min, inductor – *S.cerevisiae* culture on YPD-agar in early stationary phase (20 hour old), recipient – *S.cerevisiae* culture 15 min after inoculation. Culture density is higher at induction than in control: A –250–420 min, *P*<10^−4^; B –250–420 min, *P*<10^−2^,

Plated onto minimal medium with 0,1% glucose, yeast showed a slower (suppressed) dynamics of budding (compare control lines in fig. 4A and B). On this medium induction led to budding stimulation, the culture achieving maximal BI ∼1 hour earlier than in control (fig. 4B). Plated onto minimal acetate-containing medium, control culture showed practically no budding (BI<10%) up to 270 min after inoculation (fig. 4C), and the induced culture achieved maximal BI of ∼50%, which was 5–10 times higher than in control at the same time (see 210–270 min period in fig. 4C, *P*<10^−5^). Budding-stimulation on minimal media led also to growth stimulation (fig. 5). Still, the budding stimulation effect could also be observed even on extremely weak media lacking nitrogen, where subsequent growth was impossible (fig. 4D, growth not shown).

When on similar media, with malate, succinate or fumarate as the only substrate (instead of glucose or acetate), or with no substrate at all, the recipient culture showed no budding either in control or after induction (data not shown).

#### The recipient cultures can be stimulated only during the lag period of budding, with the induction lasting from 15 to 150 min

**Figure 6:**
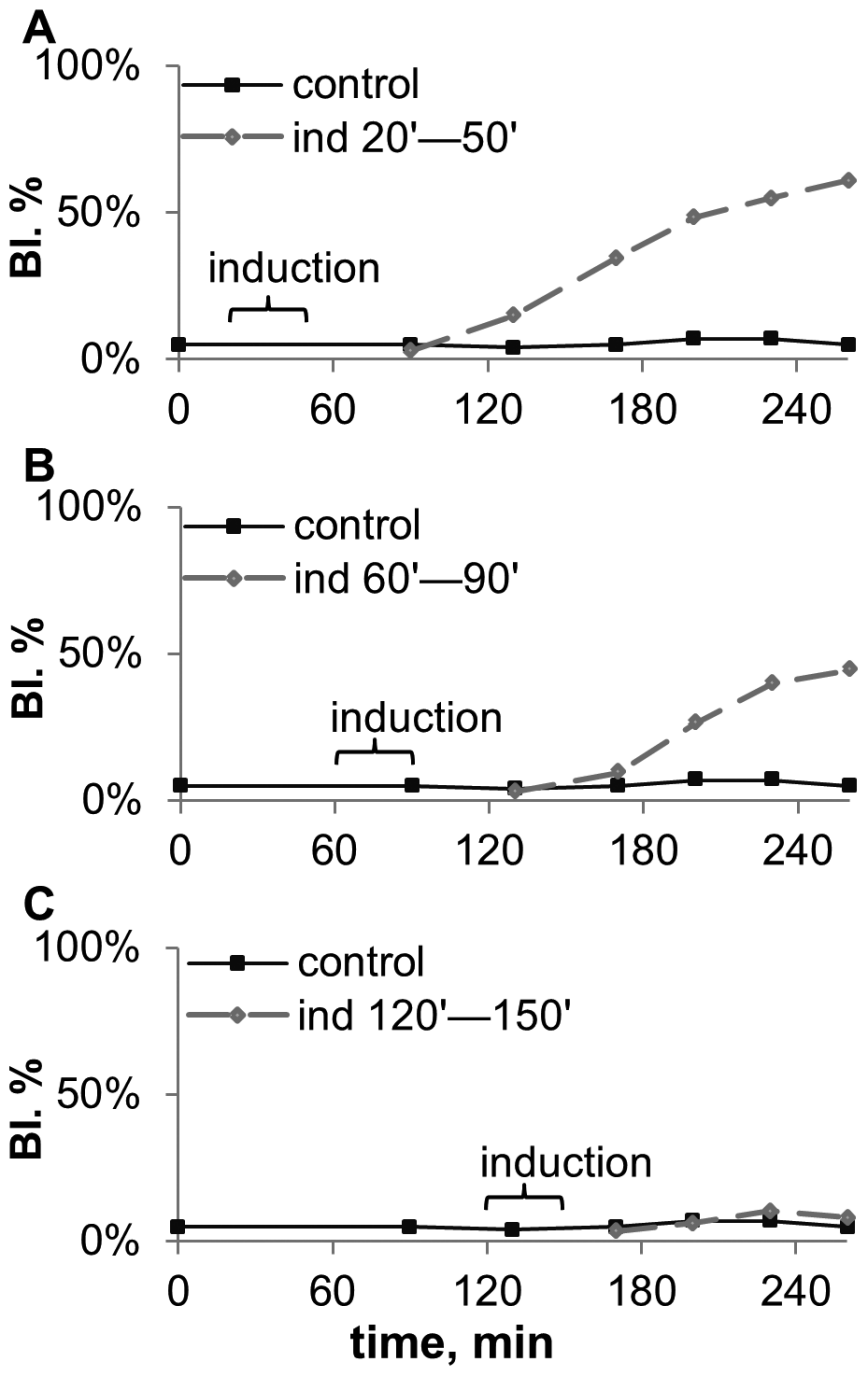
Budding dynamics of *S.cerevisiae* after 30 min induction, started at different age of the recipient. A. 20 min after inoculation, B. 60 min after inoculation, C. 120 min after inoculation. The experiment scheme is given in fig. 3A. Duration of induction was 30 min, inductor – *S.cerevisiae* culture on YPD-agar in early stationary phase (20 hour old). Medium content: CH_3_COONa 0,1%+KH_2_PO_4_ 0,1%+MgSO_4_ 0,05%+CaCl_2_ 0,01%+NaCl 0,01%, pH5,5.

The recipient cultures could be stimulated only during the first ∼2 hours after inoculation, and the earlier the recipient was subjected to induction, the higher was the observed effect (fig. 6). Notice that the effect of induction exhibited ∼2 hours after the beginning of induction, and 1–1,5 hour after its end.

The minimal length of induction that produced any budding-stimulation effect was found to be 10–15 min. The effect was increasing to maximum with the induction length rising up to 60–90 min, and remained constant for longer inductions (table 1).

**Table 1:** Budding index of agar cultures of *S.cerevisiae*, 270 min after inoculation – in control and after induction of various length (see fig. 4D for the whole budding curve on this medium).

Length of induction, min	Budding index, % (age of recipient –270 min)
control	6%±3%
3	4%±4%
10	21%±14%
30	34%±7%
60	50%±12%
90	44%±10%
150	47%±10%

The experiment scheme is given in fig. 3A. Inductor – *S.cerevisiae* culture on YPD-agar in early stationary phase (20 hour old), recipient – *S.cerevisiae* culture 15 min after inoculation.

Medium content: CH3COONa 0,1%+KH2PO4 0,1%+MgSO4 0,05%+CaCl2 0,01%+NaCl 0,01%+agar 3%, pH5,5.

#### Yeast cultures used as inductors must be alive and growing on rich media

Cultures grown on minimal media didn’t produce reliable budding stimulation effect (on any recipient cultures) with induction lasting either 30 or 90 min (data not shown). Yeast of the same strain grown on rich growth media (YPD or beer worth) produced budding stimulation effect (on proper recipient cultures) from exponential phase to the beginning of stationary phase (4–30 hours old – see sect. 4.1.1 and 4.1.2, table 2). Under these conditions, both agar and suspension cultures were good inductors. Lag-period cultures had a much lower induction capacity comparing to older inductors (table 2). Dead (boiled) cultures didn’t stimulate budding at all (data not shown).

**Table 2:** Budding index of agar cultures of *S.cerevisiae*, 210 min after inoculation – in control and after 120 min induction with various inductors (see fig. 4D for the whole budding curve on this medium).

Inductor		Budding index, % (age of recipient –210 min)
Control		5%±4%
**Agar culture**	Lag-period (15 min)	18%±13%
	Exponential phase (7 hour)	50%±12%
	Early stationary phase (20 hour)	46%±9%
**Suspension culture**	Exponential phase (7 hour)	45%±6%
	Early stationary phase (20 hour)	47%±11%

The experiment scheme is given in fig. 3A. Inductor – *S.cerevisiae* cultures of various age, in YPD (suspension) and on YPD-agar. Recipient – *S.cerevisiae* culture 15 min after inoculation.

Medium content: CH3COONa 0,1%+KH2PO4 0,1%+MgSO4 0,05%+CaCl2 0,01%+NaCl 0,01%+agar 3%, pH5,5.

### The Induction Effect is Caused by a Volatile Chemical Factor that can be Absorbed by Alkaline Solution

**Figure 7:**
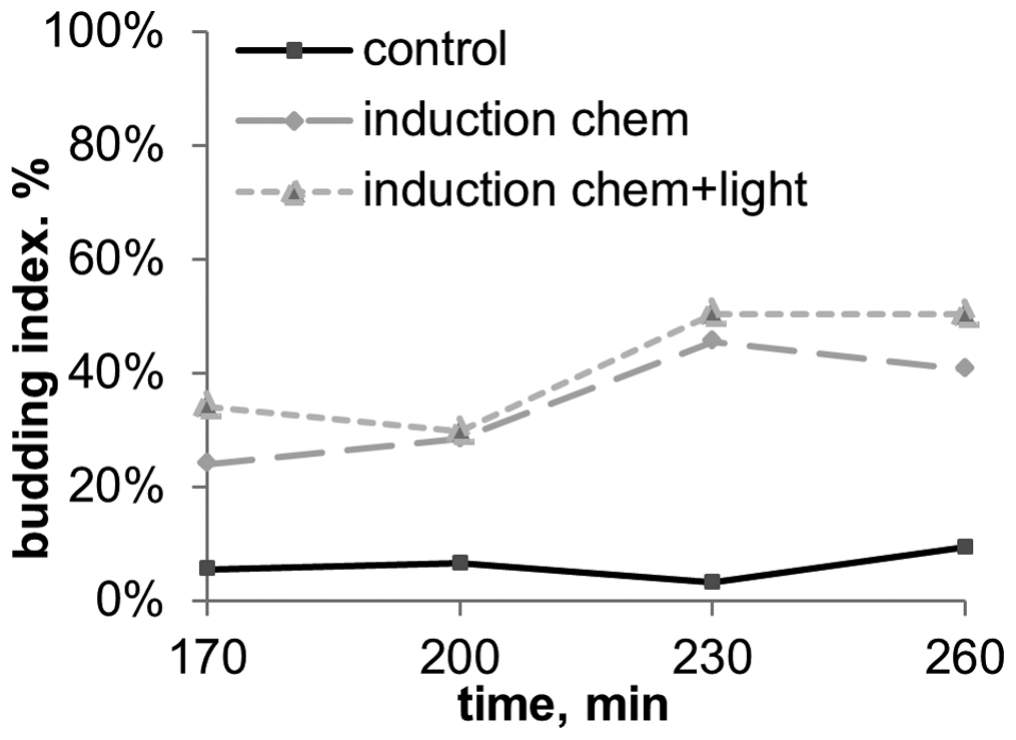
Budding dynamics of *S.cerevisiae* after 30 min induction in a big airtight container with inductor (the experiment scheme is given in fig. **3B).** **** induction chem+light – plate No 1 in fig. 3B, induction chem – plate No 2 in fig. 3b (no optical contact with the inductor). Inductor – *S.cerevisiae* culture on YPD-agar in early stationary phase (20 hour old). Medium content: CH_3_COONa 0,1%+KH_2_PO_4_ 0,1%+MgSO_4_ 0,05%+CaCl_2_ 0,01%+NaCl 0,01%, pH5,5.

To test whether the inducing factor was a volatile chemical, we separated the inductor and the recipient with metal, glass and quartz plates, and the budding-stimulating effect disappeared in any case (data not shown). The effect was also missing if the atmosphere between the inductor and the recipient was being constantly renewed during the experiment (data not shown). Two identical recipient plates fixed inside a big container with inductor, equally accessible to volatile chemicals, but oppositely located towards the inductor (fig. 3B), showed standard budding stimulation, identical for both recipient plates (fig. 7). Thus the induction effect was definitely caused by a volatile chemical factor secreted by the inductor.

**Figure 8:**
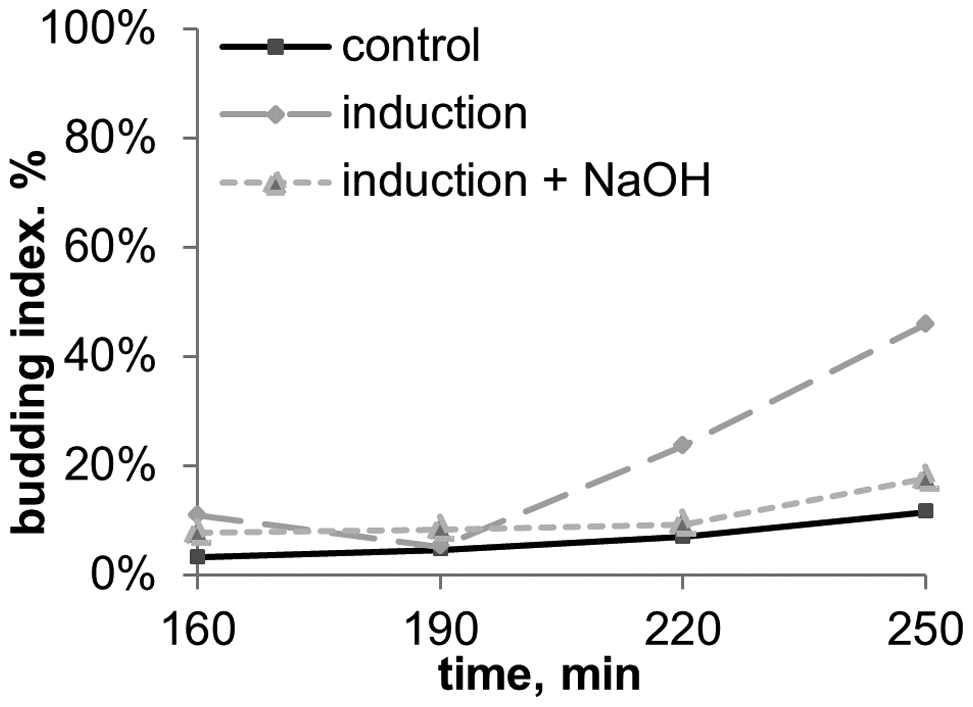
Budding dynamics of *S.cerevisiae* after 30 min induction with and without alkaline trap for acidic volatile chemicals (the experiment scheme is given in fig. **3C).** **** Inductor – *S.cerevisiae* culture on YPD-agar in early stationary phase (20 hour old). Medium content: CH3COONa 0,1%+KH2PO4 0,1%+MgSO4 0,05%+CaCl2 0,01%+NaCl 0,01%, pH5,5.

We then tested if the inducing chemical was alkaline or acidic, by fixing a small Petri dish with 1M NaOH solution inside the recipient plate, as a trap for acidic chemicals from the air (fig. 3C). Addition of this trap utterly abolished the stimulation effect at 30 min induction (fig. 8) and decreased the effect more than twice at 120 min induction (data not shown). A separate set of experiments showed that addition of such a trap (Petri dish with 1 M NaOH) didn’t influence budding or growth curves of non-stimulated (control) cultures (data not shown).

Thus the budding stimulation effect was caused by a volatile chemical factor, secreted by yeast cultures from early exponential phase to early stationary phase, and absorbed by alkaline solutions.

### Exogenous Carbon dioxide Mimics the Induction Effect

The only known chemical secreted by yeast and corresponding to all the data obtained, is CO_2_. NH_3_ and the known quorum sensing factors – tryptophol and phenilethanol – are not absorbed by alkaline solutions. Besides, the quorum sensing factors are not volatile, and NH_3_ is not secreted by yeast colonies till rather late stationary phase (4–10 days old [5]).

Measured with infrared CO_2_ sensor, the rate of CO_2_ production by our inductor cultures was found ∼0,1 micromole/sec from 1 cm^2^ of agar medium (V. Ptushenko, unpublished data). This rate of CO_2_ production leads to accumulation of ∼1% CO_2_ in 10–20 min, in the atmosphere between the recipient and the inductor. To check the inducing capacity of CO_2_, the recipient cultures were put into hermetically closed containers, and atmosphere with various concentrations of CO_2_ (0,1–4%) was created inside. Exogenous CO_2_ stimulated budding in the recipient at least in the concentrations from 0,1% to 4% (table 3), and at induction length more than 10 min (table 4). These conditions generally corresponded to the amount of CO_2_ secreted by the inductor culture.

**Table 3:** Budding index of agar cultures of *S.cerevisiae*, 270 min after inoculation – in control and after induction with CO_2_ of various concentration (see fig. 4D for the whole budding curve on this medium).

CO_2_ concentration	Budding index, %
control	5%±4%
0,13%	17%±7%
0,25%	36%±9%
0,5%	45%±16%
1%	37%±12%
2%	30%±10%
4%	25%±7%

Duration of induction –150 min.

Medium content: CH3COONa 0,1%+KH2PO4 0,1%+MgSO4 0,05%+CaCl2 0,01%+NaCl 0,01%+agar 3%, pH5,5.

**Table 4:** Budding index of agar cultures of *S.cerevisiae*, 270 min after inoculation – in control and after induction with *S.cerevisiae* culture, or with 1% exogenous CO_2_ (see fig. 4D for the whole budding curve on this medium).

Length of induction, min	Budding index, % (age of recipient –270 min)	
	Yeast induction	1% CO_2_ induction
control	6%±3%	5%±4%
3	4%±4%	7%±5%
10	21%±14%	14%±7%
30	34%±7%	23%±5%
90	44%±10%	33%±6%
150	47%±10%	37%±12%

Inductors: *S.cerevisiae* culture in early stationary phase (20 hour); 1% exogenous CO_2_. Various duration of induction.

Medium content: CH3COONa 0,1%+KH2PO4 0,1%+MgSO4 0,05%+CaCl2 0,01%+NaCl 0,01%+agar 3%, pH5,5.

Thus the effect of budding stimulation, observed in *S.cerevisiae* cultures on specific poor media, when in contact with another actively growing yeast culture, was caused by CO_2_, secreted by the latter, and exerting the stimulating influence in concentrations 0,1–4% in the atmosphere, and at the induction length ≥10 min.

### Some Evidences for Signaling Action of CO2

There can be three general mechanisms of CO_2_ action on yeast:

1. medium acidification,
2. heterotrophic fixation of CO_2_ [9], [28], [29],
3. signaling action (through adenylyl cyclase [7], [8]).

To test the first opportunity, we performed the main budding stimulation experiments on media with different pH. Budding stimulation, both by inductor yeast cultures, and exogenous CO_2_, was equally observed (on appropriate minimal media – see section 4.1.1) at pH from 4,5 to 6 (data not shown). The medium pH in the recipient culture after the end of induction was equal to pH in the control culture (and not changed comparing to initial pH of the medium). Thus, the stimulation effect was not connected to any CO_2_-induced change of the medium pH.

Metabolic CO_2_ fixation is for the greatest part taking place in reactions of phoshptryose carboxylation, generating oxaloacetate (OA) and “supporting” the Krebs cycle [30]. This way is important on media with glucose, and practically useless on media with acetate, as all OA is generated through glyoxylate bypass [30]. To test whether the budding stimulation effect was connected to metabolic CO_2_ fixation, we performed our main experiments on glucose and acetate containing minimal media (see fig. 1B and C), with addition of 0,1% oxaloacetate. This led to increase of both control and CO_2_-stimulated budding dynamics on both media (comparing to identical media without OA – see table 5), but didn’t decrease the culture sensitivity to CO_2_. Absolute increase of budding, caused by CO_2_, was equal or even slightly higher on media with OA than on identical media without OA (table 5, column ΔBI). Thus, OA was used as additional substrate, important for the culture budding (table 5) and growth (data not shown), but didn’t “substitute” exogenous CO_2_. Besides, CO_2_ action on yeast was equally high on glucose and acetate containing media, with or without additional OA.

**Table 5:** Budding index of agar cultures of *S.cerevisiae* on different media with and without oxaloacetate – in control and after induction with 4% CO_2_.

Medium	Age of recipient, min	Budding index, %		ΔBI (induction – control), %
		Control	4% CO_2_ induction	
Medium A	150	15%±6%	40%±8%	25%, *P*<10^−4^
Medium A+oxaloacetate		26%±10%	58%±7%	32%, *P*<10^−4^
Medium B	240	5%±5%	25%±7%	20%, *P*<10^−5^
Medium B+oxaloacetate		15%±5%	47%±10%	32%, *P*<10^−5^
Medium B	270	5%±4%	30%±10%	25%, *P*<10^−5^
Medium B+oxaloacetate		30%±5%	61%±10%	31%, *P*<10^−5^

Medium content:

A – Minimal medium with glucose (glucose 0,1%+(NH_4_)_2_SO_4_ 0,1%+KH_2_PO_4_ 0,1%+MgSO_4_ 0,05%+CaCl_2_ 0,01%+NaCl 0,01%, pH5,5),

B – Minimal medium with acetate, without nitrogen (CH_3_COONa 0,1%+KH_2_PO_4_ 0,1%+MgSO_4_ 0,05%+CaCl_2_ 0,01%+NaCl 0,01%, pH5,5).

Inductor –4% CO_2_, length of induction –120 min. Recipient – *S.cerevisiae* culture 15 min after inoculation.

Thus, the CO_2_-induced budding stimulation effect in our experiments was not connected to non-specific stimulation of metabolism through CO_2_ fixation, and remained as high (or even higher) on media containing excessive amount of oxaloacetate, the key product of CO_2_ fixation.

## Discussion

In the last decade a number of works appeared, showing CO_2_ sensitivity for a vast number of microorganisms [9]. Two general mechanisms of CO_2_ action on the cell are known: (1) metabolic – heterotrophic fixation, and (2) regulatory – participation in signaling pathways. Heterotrophic fixation of CO_2_, long known for *S.cerevisiae* [30], *Schizosacharomyces pombe* [29], and other species, is essential for culture growth on minimal media, mainly by supporting Krebs cycle through phosphotriose to oxaloacetate carboxylation. This way is not active when the culture is grown on rich media, or on minimal acetate-containing media, as all the needed oxaloacetate is produced in Krebs cycle (rich media) or glyoxylate bypass (acetate-containing media).

Regulatory action of CO_2_ goes through class IIIb (soluble or cytoplasmic) adenylyl cyclases by direct binding with their catalytic domain. This way is shown for mammals, cyanobacteria [7], and pathogenic fungi [25], in which it stimulates cell differentiation and virulence [31]. Regulatory pathway of CO_2_ sensitivity was also supposed for *S. pombe* [29], and argued for *S.cerevisiae* [26], but disproved by the same authors in [32], where they showed that the effect of HCO_3_^–^ stimulated spore formation, observed in their work, was caused by alkalization of the medium [33].

The present work is the first to show significant effects of CO_2_- mediated interaction of cells on *S.cerevisiae*. We cannot make any direct statements concerning mechanisms of our effect yet. Still we can conclude that (1) it is not connected to the medium pH shift, and (2) it is not connected to heterotrophic fixation and metabolic use of CO_2_. This allows us to suppose the budding-stimulation effect going through regulatory, rather than metabolic pathways. Besides, the effect is observed 1,5–2 hour later than the interaction is finished.

The main difference of our results from the effects of CO_2_ action, known for pathogenic fungi, is that here CO_2_ increase stimulates cell division, rather than mitosis block and cell differentiation [9], [31].

Anyway, the effect of distant CO_2_-mediated interaction of *S. cerevisiae* cultures, shown in this work, can be interpreted as cell-cell interaction, regulating cell behavior according to the culture density, i.e. a quorum sensing effect.
